# Closed-Cell Stent-Assisted Coiling of Intracranial Aneurysms: Evaluation of Changes in Vascular Geometry Using Digital Subtraction Angiography

**DOI:** 10.1371/journal.pone.0153403

**Published:** 2016-04-13

**Authors:** Ebba Beller, David Klopp, Jens Göttler, Johannes Kaesmacher, Claus Zimmer, Jan S. Kirschke, Sascha Prothmann

**Affiliations:** 1 Abteilung für Diagnostische und Interventionelle Neuroradiologie, Klinikum rechts der Isar, Technische Universität München, Munich, Germany; 2 Acandis GmbH & Co. KG, Pforzheim, Germany; University of Louisville, UNITED STATES

## Abstract

**Background:**

Stent-assisted coil embolization (SACE) plays an important role in the treatment of intracranial aneurysms. The purpose of this study was to investigate geometrical changes caused by closed-cell design stents in bifurcation and sidewall aneurysms.

**Methods:**

31 patients with 34 aneurysms underwent SACE with closed-cell design stents. Inflow angle α, determined by aneurysm neck and afferent vessel, and angle between afferent and efferent vessel close to (δ_1_), respectively, more remote from the aneurysm neck (δ_2_) were graphically determined in 2D angiography projections.

**Results:**

Stent assisted coiling resulted in a significant increase of all three angles from a mean value (±SEM) of α = 119° (±6.5°) pretreatment to 130° (±6.6°) posttreatment (P ≤ .001), δ_1_ = 129° (±6.4°) to 139° (±6.1°), (P ≤ .001) and δ_2_ = 115° (±8.4°) to 126° (±7.5°), (P ≤ .01). Angular change of δ_1_ in AcomA aneurysms was significant greater compared to sidewall aneurysms (26°±4.9° versus 8°± 2.3°, P ≤ .05). The initial angle of δ_1_ and δ_2_ revealed a significantly inverse relationship to the angle increase (δ_1_: r = -0.41, P ≤ .05 and δ_2_: r = -0.47, P ≤ .01). Moreover, angle δ_1_ was significantly higher in unruptured compared to ruptured aneurysms (135°±7.1° versus 103°±10.8°, P ≤ .05).

**Conclusion:**

Stent deployment modulates the geometry of the aneurysm-vessel complex, which may lead to favorable hemodynamic changes more similar to unruptured than to ruptured aneurysms. Our findings also suggest that the more acute-angled aneurysm-vessel anatomy, the larger the angular change. Further studies are needed to investigate whether these changes improve the clinical outcome.

## Introduction

Stent-assisted coil embolization (SACE) is a well-established endovascular therapy of cerebral aneurysms with wide necks or low dome-to-neck ratios [1–3]. Advantages of SACE over coiling alone are not only mechanical characteristics of the stent, which prevent coil prolapse and allow higher packing density, but also potential hemodynamic effects by diverting the blood flow [4–7]. Besides direct reduction of flow by the stent struts, one of the relevant hemodynamic effects by stent placement seems to be straightening of vessels-aneurysm complex [8]. Especially stents with closed-cell design show this capability of angular remodeling. The closed-cell design makes the stent work as a whole body, immediately transmitting a force used at one end to the other end [9,10]. This leads to effective straightening of a curved vessel in which the stent is implemented [9–11]. Stents with open-cell design show significantly less angular remodeling [11], however, conform better to curved vascular segments by having several independent segments instead [9,10].

Originally, stents have been designed to treat sidewall aneurysms, but it has been shown that stent implementation is also suitable for many bifurcation aneurysms [9]. Advances in SACE have led to a variety of different stent-placement methods, also allowing treatment of a subset of wide-necked aneurysms not amenable to reconstruction with a single-stent due to anatomical conformation. These methods include for example “crossing Y-stent technique”, indicating that a second stent is advanced through the first stent interstices and into the contralateral branch vessel [9,12]. Thus a variety of complex aneurysm can be treated with SACE. Previous studies even suggest stent treatment without coiling, already changing hemodynamics sufficiently enough to induce intra-aneurysmal thrombosis [13–15]. Zenteno et al attributed this therapeutic effect to the straightening of the vessel-aneurysm complex induced by balloon inflation during balloon-mounted stent deployment [11,13]. Results of computational flow and ideal aneurysm model analysis also suggest that the straightening of the parent vessel is associated with a favorable outcome [16,17], as it leads to smaller pressure, inflow volume rate and inflow velocity in the aneurysm [16,18]. To investigate the effect of closed-cell stent remodeling in real patients in a clinical setting, we measured structural changes of the vessel–aneurysm complex of bifurcation and sidewall aneurysms treated at our institution.

## Materials and Methods

### Patient Selection

The study was approved by the ethical committee of the Technische Universität, München. Patient records and information were anonymized and de-identified prior to analysis. We retrospectively identified 50 patients with 57 intracranial aneurysms treated by stent-assisted coiling with closed-cell design stents between June 2008 and July 2015. Patients with no recorded follow-up angiography (n = 13), complication due to dislocation of the proximal part of the stent into the aneurysm (n = 1), postoperative in-stent thrombosis (n = 1) or in-stent occlusion (n = 2) were excluded. No measurement of angular remodeling was possible due to insufficient image quality for the construction of the auxiliary elements in two cases, which were also excluded. The final study population included 31 patients with 34 aneurysms.

### Endovascular Treatment

Patients scheduled for elective stent-assisted coiling received oral acetylsalicylic acid (100 mg) and oral clopidogrel (75 mg) for at least 5 days before the procedure. On the day of the procedure platelet function was tested with Multiplate ® Analzyer (Roche Diagnostics, Mannheim, Germany). In case of clopidogrel resistance, clopidogrel was replaced by prasugrel with a loading dose of 60 mg and the procedure was performed the next day. All patients were kept on a regimen of both acetylsalicylic acid and clopidogrel for 3 months, after which clopidogrel was no longer given

29 aneurysms were treated with single stent-assisted coiling of which 25 were treated with Enterprise stents (Codman & Shurtleff, Inc., Raynham, MA, USA) and of which four aneurysms were treated with Acandis Acclino stents (Acandis, Pforzheim, Germany). One aneurysm was treated with one Enterprise stent and one Solitaire AB stent (Covidien, Irvine, CA, USA), due to distal displacement of the Enterprise stent. From the total amount of 24 bifurcation aneurysms, four were treated in Y-configuration: Two basilar artery (BA) bifurcation aneurysms and one anterior communicating artery (AcomA) aneurysm were treated by placing two Enterprise stents in Y-configuration; One BA bifurcation aneurysm was treated with one Acandis Acclino stent and one Leo stent (Balt, Montmorency, France) in Y-configuration. As Leo stents are made by wire braiding while Enterprise, Solitaire AB and Acandis Acclino stents are laser-cut from nitinol hypotube, the angle formed by the Leo stent was excluded. The Solitaire stent had a diameter of 4 mm, all Acclino and Enterprise stents had a diameter of 4.5 mm.

### Vascular Measurements

**Fig 1:**
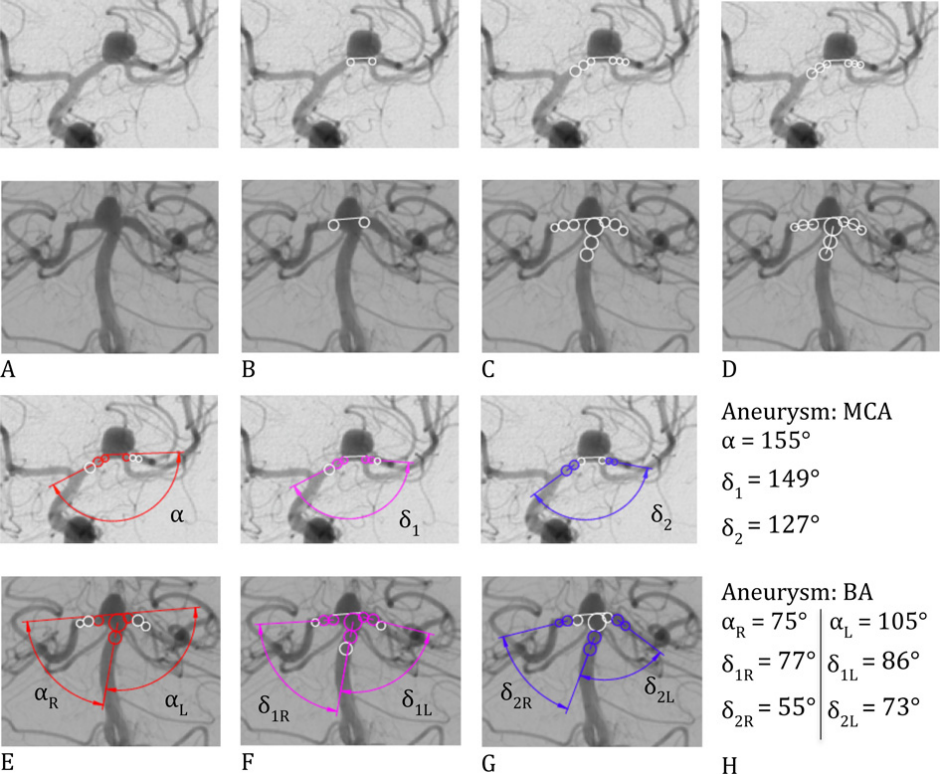
Construction of the auxiliary elements and angular measurements in sidewall and bifurcation aneurysms. Inflow angle α was determined by the aneurysm neck section line and the centerline of the afferent vessel close to the aneurysm. The angle close to the aneurysm neck (δ_1_) and more remote from the aneurysm neck (δ_2_) were determined by centerlines of the efferent and afferent vessel close (δ_1_), respectively, located more remotely from the aneurysm neck (δ_2_).

DSA was performed on a bi-planar Philips Allura Xper FD (Philips Medical Systems B.V., Best, The Netherlands). For assessment of the vessel-aneurysm complex, standard 2D DSA projections angled perpendicular to the longest axis of the aneurysm with the best view of aneurysm sac and neck were analyzed before and after stent-assisted coiling. For graphical measurements of the vascular angles we additionally constructed auxiliary elements with Siemens Solid Edge 2D Drafting as follows (Fig 1). Two circles adjacent to the aneurysm neck were constructed in the efferent and afferent vessel in sidewall aneurysms, respectively, in both efferent vessels in bifurcation aneurysms, with the same diameter as the vessel they were positioned in. An aneurysm neck section line tangential to both circles was drawn. (Fig 1B) A cycle tangential to the aneurysm neck section line and to the afferent vessel was added in bifurcation aneurysms but not in sidewall aneurysms (Fig 1C, lower row). Two more cycles, again with the same diameter of the vessels in which they were positioned in, were added tangentially to each of the previously constructed cycles (Fig 1C). Centerlines were drawn between the centers of the adjacent cycles (Fig 1D). Inflow angle α was determined by the aneurysm neck section line and the centerline of the afferent vessel close to the aneurysm (Fig 1E). The angle close to the aneurysm neck (δ_1_) and more remote from the aneurysm neck (δ_2_) were determined by centerlines of the efferent and afferent vessel close (δ_1_), respectively, located more remotely from the aneurysm neck (δ_2_) (Fig 1F and 1G). Two neuroradiologists (EB, SP) evaluated vascular measurements of all aneurysms in consensus reading.

### Statistical Analysis

Differences were analyzed using two-tailed Wilcoxon signed rank test or two-tailed Mann Whitney test. Comparisons between three or more groups were analyzed by Kruskal-Wallis (KW) test with post-hoc Dunn’s test. Correlation was tested by Pearson’s correlation analysis in normally distributed data (prestenting angle) and by Spearman correlation in non-normally distributed data (vessel diameter). Data is presented as means ± SEM. Statistical analysis was performed using GraphPad Prism software (version 5.0a); p-values ≤0.05 were considered significant.

## Results

### Patient and Aneurysm Characteristics

The majority of the patients were women (74.2%) and the median age at stent implementation was 54 years (inter quartile range [IQR]: 44–64 years). Patients presented with subarachnoid hemorrhage in 11 cases (35%). Bifurcation aneurysms (n = 21) were at the following locations: BA (n = 6), middle cerebral artery (MCA) (n = 5) or AcomA (n = 10). Sidewall aneurysms (SWA) (n = 13) were found at the following locations: BA (n = 2), MCA (n = 1), posterior inferior cerebellar artery (PICA) (n = 1), superior cerebellar artery (SCA) (n = 1) or internal carotid artery (ICA): ophthalmic segment (n = 2) and communicating segment (n = 6). Aneurysm size defined as maximum diameter ranged from 1,5 to 20 mm (median: 7 mm). The size of the aneurysm neck ranged from 1 to 8mm (median: 4mm) with 4 missing values due to absence of calibration. Adjunctive stenting was performed either to treat wide neck aneurysms (n = 18), aneurysm recurrence (n = 8) or due to prolapsed coil (n = 8). No intraprocedural aneurysm rupture occurred during the stent-coiling procedures in any of the patients.

### Follow-Up Analysis

For assessment of vessel anatomy, matching 2D projections of the angiograms before and after stent-assisted coiling were analyzed with a median follow-up period of seven months (IQR: 4–11 months). Auxiliary elements for measurement of angle δ_2_ could not be constructed in five cases. This was due to the vessel anatomy remote from the aneurysm neck with the course of the vessel orthogonal to the image plane. The diameter of the afferent and efferent vessel was calculated after correction of estimated radiographic magnification factor on digital subtraction angiography. Missing values were due to absence of calibration (no calibration of DSA projections pretreatment = 19, posttreatment = 5), one patient was excluded who developed vasospasm after subarachnoidal bleeding and was treated with nimodipine during follow-up angiography, respectively.

### Effective Straightening of the Aneurysm-Vessel Complex after Stent Placement

**Fig 2:**
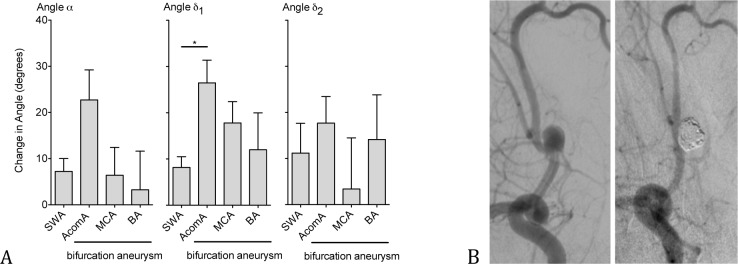
Effective straightening of the aneurysm-vessel complex after stent placement. Angular differences of sidewall and bifurcation aneurysms pre- and posttreatment (A). DSA-Angiography of a 41-year old patient with AcomA aneurysm, showing an effective straightening of the aneurysm-vessel complex 23 months after stent-assisted coiling (B), (graph shows mean ± SEM, *: P ≤ .05, for n-values see Table 1).

Changes in all three angles α, δ_1_ and δ_2_ were detected after stent assisted coiling, suggesting that stent deployment led to effective straightening of the affected vessel. Stent assisted coiling resulted in a highly significant increase of the inflow angle α from a mean value (SEM) of 119° (±6.5°) pretreatment to 130° (±6.6°) posttreatment (P ≤ .001). Analysis of δ_1_ and δ_2_, measuring the angle close to the aneurysm neck or more remote from the aneurysm neck respectively, also showed a significant increase of δ_1_ from a pretreatment value of 129° (±6.4°) to 139° (±6.1°) posttreatment (P ≤ .001) and of δ_2_ with a pretreatment value of 129° (±6.4°) compared to 139° (±6.1°) posttreatment (P ≤ .01) (Table 1). The Kruskal-Wallis test with post-hoc Dunn's multiple comparison test showed a significant greater angular change of δ_1_ in AcomA aneurysms compared to sidewall aneurysms (26°±4.9° versus 8°± 2.3°, P ≤ .05), but no significant change of angle α and δ_2_. KW test on comparison for angular change between different bifurcation aneurysms (AcomA, MCA and BA) also failed to show significant differences among them (Fig 2).

**Table 1:** Angle α, δ_1_ and δ_2_ before and after stent-assisted coiling.

Location of the Angle	Prestenting Angle			Post-Stent-Coiling Angle		
	α	δ_1_	δ_2_	α	δ_1_	δ_2_
Total	119.2±6.5	129.0±6.4	115.0±8.4	130.1±6.6	139.0±6.1	126.2±7.5
	(n = 37)	(n = 37)	(n = 32)	***	***	**
Sidewall	134.8±12.2	145.4±10.8	128.7±14.3	142.0±12.5	153.8±10.5	134.2±14.2
	(n = 13)	(n = 13)	(n = 10)	*	**	ns
Bifurcation	110.8±7.2	111.6±7.0	109.7±10.3	123.6±7.5	131.4±7.2	123.0±8.9
	(n = 24)	(n = 24)	(n = 22)	**	***	*
AcomA	115.8±10.6	124.4±9.6	126.2±14.4	138.5±10.4	150.8±9.9	145.4±14.5
Bifurcation	(n = 11)	(n = 11)	(n = 10)	*	**	**
MCA	141.6±11.6	117.6±16.3	118.8±18.4	148.0±11.9	135.4±16.4	122.2±11.9
Bifurcation	(n = 5)	(n = 5)	(n = 4)	ns	ns	ns
BA	84.6±6.5	90.3±10.4	74.8±12.7	87.8±2.9	102.3±5.6	88.9±7.1
Bifurcation	(n = 8)	(n = 8)	(n = 8)	ns	ns	ns

The p-value applies to the prestenting angle versus the respective post-stent-coiling angle (ns: P>.05, *: P ≤ .05, **: P ≤ .01, ***: P ≤ .001). Prestenting angles have the same number (n) of angle values as post-stent-coiling angles (table shows mean ± SEM).

### Relationship between Pretreatment Angle or Vessel Diameter and Angular Change

**Fig 3:**
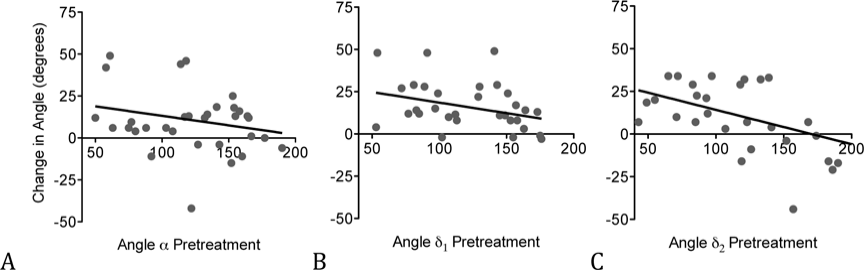
Inverse relationship between the angular change and the pretreatment angle (α, δ_1_ and δ_2_). α: n = 37, r = -0.26, P = 0.14 (A), δ_1_: n = 37, r = -0.41, P ≤ .05 (B) and δ_2_: n = 32, r = -0.47, P ≤ .01 (C), x axis, angle α, δ_1_ or δ_2_ before treatment, y axis, angular difference between pre- and posttreatment.

We hypothesized that the degree of angular remodeling was dependent on the angle of the vessel segment and on the vessel diameter, each before treatment. Analysis of the angular change pre- and posttreatment and the pretreatment angle revealed a significantly inverse relationship of angle δ_1_ and δ_2_ (δ_1_: r = -0.41, P ≤ .05 and δ_2_: r = -0.47, P ≤ .01). There was no significant relationship of the angular change and the prestenting angle of inflow angle α (r = -0.26, P = 0.14) (Fig 3). Analysis of the angular change and the presenting diameter of the proximal (α: r = -0.50, P = 0.06, δ_1_: r = -0.05, P = 0.86 and δ_2_: r = 0.29, P = 0.33) and distal vessel (α: r = -0.26, P = 0.38, δ_1_: r = -0.46, P = 0.11 and δ_2_: r = 0.07, P = 0.83) did not show a significant relationship either (n = 18 for α and δ_1_, n = 16 for δ_2_).

### Change in Vessel Diameter of the Afferent and Efferent Vessel After Stent Deployment

**Fig 4:**
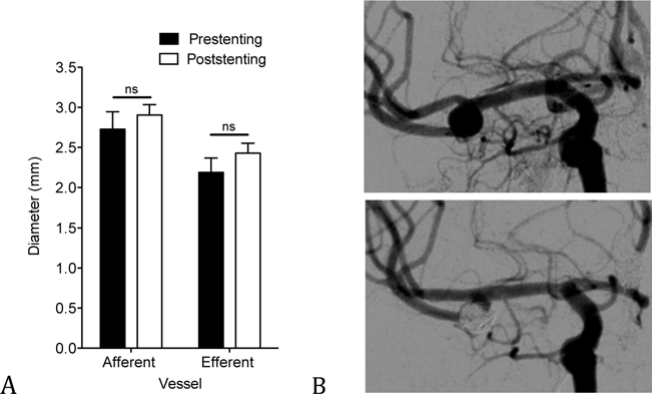
Slight yet not significant increase of the vessel diameter after stenting. Change in vessel diameter of afferent and efferent vessel pre- and posttreatment, n = 18 for the presenting group and n = 30 for the poststenting group (graphs shows mean ± SEM) (A). DSA-angiography of a 43-year old patient with MCA aneurysm showing an increase in diameter especially of the efferent vessel 11 months after SACE (B).

The diameter of the proximal (afferent) vessel and the distal (efferent) branch showed a slight yet not significant trend towards an increase from 2.73mm (±0.2mm) pretreatment to 2.90mm (±0.1mm) posttreatment (P = 0.18), and from 2.19mm (±0.2mm) to 2.43mm (±0,1mm), (P = 0.25), respectively (Fig 4).

### Angle Formation and Vessel Diameter in Ruptured Aneurysms Compared to Unruptured Aneurysms

**Fig 5:**
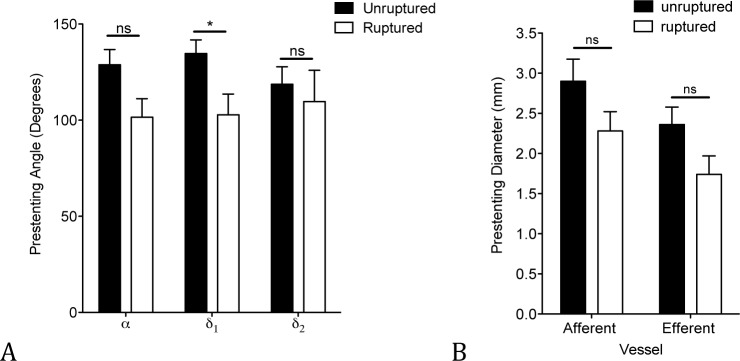
Aneurysm-vessel complex in ruptured aneurysms compared to unruptured aneurysms. Angle of unruptered (α: n = 24, δ_1_: n = 24 and δ_2_: n = 19) and ruptured aneurysms (n = 13) before treatment (graph shows mean ± SEM, *: P ≤ .05), (A). Vessel size of unruptured (n = 13) and ruptured aneurysms (n = 5) (B).

Before treatment, angle δ_1_ was significantly higher in the unruptured group at 135°±7.1° compared to 103°±10.8° for the ruptured group (P ≤ .05). Inflow angle α showed greater values in the unruptured subset (138°±8.0°) compared to the ruptured subset (102°±9.7°), almost reaching statistical significance (P = 0.0503). There was no statistically significant difference of angle δ_2_ between unruptured aneurysms (119°±9.1°) and ruptured aneurysm (110°±16.3°), (P = 0.28) (Fig 5A). Comparing the diameter of unruptured to ruptured aneurysms, we found a slight trend that did not reach statistical significance towards a greater diameter in the unruptured group compared to the ruptured group. The diameter of the afferent vessel of unruptured aneurysms had a mean value of 2.90mm (±0.28mm) compared to 2.28mm (±0.24mm) of ruptured aneurysms (P = 0.15) and the diameter of the efferent vessel of unruptured aneurysms had a mean value of 2.36mm (±0.22mm) compared to 1.74mm (±0.23mm) of ruptured aneurysms (P = 0.14) (Fig 5B).

## Discussion

In our study, stent-assisted coil embolization led to effective straightening of the aneurysm-vessel complex and a slight trend towards an increasing vessel diameter. These geometrical changes in SACE are likely to contribute to favorable hemodynamics within the aneurysm.

Hemodynamic factors play an important role in the pathogenesis, progression, and rupture of cerebral aneurysms and are especially sensitive to variations in vessel geometry [16,19–21]. A previous study that used ideal intracranial aneurysm models found a strong dependence of aneurysm hemodynamics on the curvature of the parent artery; the straighter the parent vessel, the smaller the pressure, inflow volume rate and inflow velocity in the aneurysm [16]. Reduction of blood flow into the aneurysm and of the flow velocity magnitude at the neck were found to be related to thrombus formation, supporting a complete occlusion of the aneurysm and a favorable outcome after endovascular treatment [16,17]. Similar results were reported in a study about the hemodynamic effect of changes in key geometrical properties of the aneurysms, using computational fluid dynamics (CFD). After changing a straight parent vessel into a curved one, they observed a drastic change in blood flow dynamics, with an average velocity about ten times higher in the aneurysm sac [18]. Further CFD analysis by Gao et al showed that the bifurcation angle determines the hemodynamic environment of the aneurysm following SACE by changing pressure and wall shear stress in a favorable direction. They could also show that angular remodeling is more significant immediately after Y-stenting and during the first six months of follow-up. Then, a steady state is seen because the potential energy of the stent is mostly released [22,23]. All in all straightening of the aneurysm-vessel complex suggests a favorable hemodynamic change within the aneurysm.

Previous studies mainly focused on vascular remodeling of either bifurcation aneurysms [11,24,25] or sidewall aneurysms only [8,26]. In this study we compared bifurcation to sidewall aneurysms, demonstrating that stent-assisted coiling led to a greater change in the angle of bifurcation aneurysms of the anterior communicating artery compared to sidewall aneurysms. Interestingly sidewall aneurysms, especially of the ACI, compared to bifurcation aneurysm, such as AcomA, have a less acute angle formation before treatment and a greater diameter of the parent vessel. This finding might suggest that the larger the pre-stenting angle and the larger the vessel size, the smaller the angular change of δ_1_, δ_2_ and also inflow angle α [11]. Other studies on angle remodeling of bifurcation aneurysm after SACE also found this inverse relation between extent of vascular modification and vessel diameter or pretreatment angle, latter corresponding with angle δ_1_ of our study [11,24]. In our study we could only show a significant inverse relationship between angular change and presenting angle for angle δ_1_ and δ_2_ but not between angular change and vessel diameter. The latter could be due to the fact that we had to accept missing values of the vessel diameter (n = 19) because of the absence of calibration.

Previous studies on vascular remodeling effects of SACE have focused on straightening of the parent vessel [20,22,24,25]. In our study we could show that SACE did not only lead to effective straightening but also showed a trend towards an increasing diameter of the parent vessel, suggesting not only a bending [11] but also a radial force exerted by the deployed stent. Studies have shown that specific locations on the cerebral vasculature, e.g. AcomA or distal vessels, have a higher percentage of small ruptured aneurysms compared to other more proximal locations, such as ICA, suggesting a higher rupture risk for aneurysms of vessels with small calibers and lower risks for larger-diameter vessels, independent from aneurysm size [27,28]. In an aneurysm flow dynamics study, Tremmel et al demonstrated that only reducing the diameter of the vessel parent, while keeping all other morphological parameters unchanged, already leads to considerable change in flow patterns to more complex flow structures with multiple associated vortices and an increase of the area exposed to low wall shear stress (<0.5 Pa) [29]. Large areas with low wall shear stress on the aneurysm inner wall, which are known to trigger atherosclerotic and inflammatory pathways [21,30,31], as well as complex flow patterns, have been identified to correlate with aneurysm growth [32,33] and rupture [29,30]. Thus, increasing the diameter of the parent vessel and by that decreasing hemodynamic risk factors for aneurysm rupture could be an additional positive effect of SACE.

Comparing ruptured to unruptured aneurysms, ruptured aneurysms seem to have a more acute-angled vessel configuration and a smaller vessel diameter than unruptured aneurysms and by that presumably presenting a high-risk vascular configuration. Previous studies on differences in aneurysm characteristics between ruptured and unruptured aneurysms regarding aneurysm flow angles showed conflicting results [34–37]. Inconsistent results comparing geometric and morphological aneurysm characteristics between patients with ruptured aneurysms and unruptured aneurysms could be due to lack of adjustment for patient specific risk factors for aneurysm rupture or use of different imaging techniques and measurement methodology [37,38]. Moreover, although there is no study about the change of aneurysm-vessel configurations after rupture, it is possible that the event of rupture itself leads to structural remodeling.

The results of our study should be viewed in consideration of its limitations. This was a retrospective study with a relatively small sample size of 31 patients with 34 aneurysms. Further studies with a larger number of patients from multiple centers will be required to verify the findings. This especially applies to the changes in vessel diameter after SACE, as we had to accept missing values due to absence of calibration. Moreover, the sample size of subgroups comparing Enterprise stents with Acandis Acclino stents or Y-configuration with single-stents were too small for statistical analysis. Our hypothesis that increasing the diameter and straightening of the parent vessel contributes to favorable hemodynamics within the aneurysm is supported by previous computational fluid dynamics studies [18,22,29] but has to be corroborated with clinical results and with long-term follow-up angiographic outcomes focusing on recanalization rates.

For graphic analysis of the different angles we used freely available software (Siemens Solid Edge 2D Drafting) and 2D working projections (DSA), which can be easily applied in clinical practice. Although, attention should be paid when performing angle measurements because of large variabilities depending on the viewing angle. As our standard procedure includes the acquisition of a projection perpendicular to the aneurysm, usually by using a 3D DSA-angiography, in order to plan the most effective treatment, we could minimize measurement errors. 3D projections before and after treatment would be preferable for more accurate measurements but were less commonly performed for follow up imaging in our clinical practice.

In conclusion, stent-assisted coil embolization leads to effective straightening of the aneurysm-vessel complex and a trend towards an increase in the diameter of the afferent vessel. These geometrical changes in SACE are likely to contribute to favorable hemodynamics within the aneurysm and are more similar to unruptured than to ruptured aneruysms. Further studies are warranted to investigate whether these changes are associated with a favorable outcome of the patient.

## Abbreviations

SACE: Stent-assisted coil embolization
SWA: sidewall aneurysm
AcomA: anterior communicating artery
BA: basilar artery
MCA: middle cerebral artery
PICA: posterior inferior cerebellar artery
SCA: superior cerebellar artery
ICA: internal carotid artery
d: diameter
SEM: standard error of mean
